# Effects of Consecutive Versus Non-consecutive Days of Resistance Training on Strength, Body Composition, and Red Blood Cells

**DOI:** 10.3389/fphys.2018.00725

**Published:** 2018-06-18

**Authors:** Yifan Yang, Pang B. Bay, Yongtai R. Wang, Junli Huang, Hilary W. J. Teo, Jorming Goh

**Affiliations:** ^1^Physical Education and Sports Science, National Institute of Education, Nanyang Technological University, Singapore, Singapore; ^2^Combat Protection and Performance Program, DSO National Laboratories, Singapore, Singapore; ^3^Department of Physiology, National University of Singapore, Singapore, Singapore

**Keywords:** resistance training, recovery period, muscle strength, muscle mass, bone mineral density, fat loss, erythrocytes, hematology

## Abstract

Health authorities worldwide recommend 2–3 days per week of resistance training (RT) performed ∼48–72 h apart. However, the influence of recovery period between RT sessions on muscle strength, body composition, and red blood cells (RBCs) are unclear.

**Aim:** Examine the effects of three consecutive (C) or non-consecutive (NC) days of RT per week for 12 weeks on strength, body composition, and RBCs.

**Methods:** Thirty young, healthy and recreationally active males were randomly assigned to 3 C (∼24 h between sessions) or NC (∼48–72 h between sessions) days of RT per week for 12 weeks. Both groups performed three sets of 10 repetitions at 10-repetition maximum (RM) of leg press, latissimus pulldown, leg curl, shoulder press, and leg extension for each session. Ten RM and body composition were assessed pre- and post-RT. RBC parameters were measured on the first session before RT, and 0 and 24 h post-3rd session in untrained (week 1) and trained (week 12) states.

**Results:** No training × group interaction was found for all strength and body composition parameters (*p* = 0.075–0.974). Training increased strength for all exercises, bone mineral density, and total body mass via increased lean and bone mass (*p* < 0.001). There was no interaction (*p* = 0.076–0.994) and RT induced temporal changes in all RBC parameters (*p* < 0.001–0.003) except RBC corrected for plasma volume changes (time × training interaction; *p* = 0.001). Training increased hematocrit and lowered mean corpuscular hemoglobin and mean corpuscular hemoglobin concentration (*p* = 0.001–0.041) but did not alter uncorrected RBC, hemoglobin, mean corpuscular volume and RBC distribution width (*p* = 0.178–0.797).

**Conclusion:** Both C and NC RT induced similar improvements in strength and body composition, and changes in RBC parameters.

## Introduction

Health authorities worldwide, such as the American College of Sports Medicine (ACSM; Garber et al., 2011) and World Health Organization (World Health Organization, 2010), recommend that adults perform RT at least twice a week for health benefits. The recommendation is to perform the RT sessions at least 48 h apart, i.e., on NC days (Garber et al., 2011). This recommendation stems from two acute studies by Haddad and Adams (2002) and Bickel et al. (2005) which demonstrated that a recovery period of 48–72 h between RT sessions is needed to optimize the molecular responses favorable to gains in muscle size and strength based on two bouts of isometric contraction via electrical stimulation in rats (Haddad and Adams, 2002) and humans (Bickel et al., 2005).

However, the chronic influence of recovery period between RT sessions on muscle strength and mass is unclear. In addition, it is common for fitness enthusiasts, serious athletes (including competitive weightlifters) and “weekend warriors” to perform RT on C days, and yet studies on C days of RT are scarce. One recent study showed that 37 C days of high intensity squatting increased (based on values with no significance testing) the 1RM for squat in two male powerlifters and one male weightlifter, with the peak 1RM (tested daily except day 36) occurring on day 35 or 37 (Zourdos et al., 2016). Fat-free mass also increased among all participants but changes in fat mass and quadriceps thickness were inconsistent. Another subsequent study of five resistance-trained men showed that 21 C days of 1RM testing and maximal voluntary isometric contraction of the elbow flexors significantly improved the 1RM strength in both arms to a similar extent, and that the arm that performed three additional sets of elbow flexions daily also significantly increased arm muscle thickness (Dankel et al., 2017). These results suggest that prolonged C days of RT can improve muscle strength and size in individuals that are very experienced in RT, but it remains to be investigated if the results would be applicable to the general population, and if C days of RT would produce differential responses to NC days of RT, given the small sample sizes in these studies and lack of direct comparison between C and NC days of RT. Only one recent study compared C and NC days of RT and showed that 3 C (*n* = 10) or NC (*n* = 11) days of RT per week for 7 weeks produced similar adaptations in maximum strength and skinfold-determined body composition (Carvalho and Rodrigues Santos, 2016). However, the authors only reported strength changes for two exercises and did not account for the other exercises that were also performed.

Furthermore, both acute and chronic effects of recovery period between RT sessions on many other physiological variables, including RBCs, are unknown. Aside from erythrocytes’ role in athletic performance, collectively, RBC parameters are also commonly evaluated as part of a broad health screening for hematologic conditions, such as anemia. Altered RBC count may lead to fatigue, shortness of breath and other symptoms. AT in healthy population acutely decreases PV leading to a transient increase in Hct post-exercise but stimulates PV and RBC volume expansion over time (Hu and Lin, 2012; Mairbäurl, 2013). As PV expands more rapidly or greater than RBC volume, decreased RBC, Hct or Hb due to hemodilution, termed “sports anemia,” can be observed with AT. Thus, it is important to understand both acute and chronic exercise-induced changes to help differentiate between exercise-induced and pathological changes in RBC parameters. However, compared to AT, studies on RT-induced RBC changes are relatively limited (Hu and Lin, 2012), with a mix of acute and chronic RT studies that reported conflicting results on RBC parameters (Schobersberger et al., 1990; McCarthy et al., 1997; Kilgore et al., 2002; Ahmadizad and El-Sayed, 2005; Ahmadizad et al., 2006; Craig et al., 2008; Hu et al., 2008; Cakir-Atabek et al., 2009; Hulmi et al., 2010; Kilic-Toprak et al., 2012; Teixeira et al., 2014). Moreover, only two studies (Cakir-Atabek et al., 2009; Kilic-Toprak et al., 2012) investigated both acute and chronic effects of RT on RBC parameters within the same study. Furthermore, little is known about the effects of multiple bouts of RT, which is more aligned with weekly physical activity guidelines, and the 24 h recovery period on RBC to elucidate if it may be suboptimal. RBC, Hct and Hb returned to baseline levels by 30 min post-RT (Ahmadizad and El-Sayed, 2005; Ahmadizad et al., 2006; Teixeira et al., 2014); thus, it is likely that a 24 h recovery period is sufficient for RBC.

Therefore, given the above reasons, the aim of this study was to determine the effects of 3 C or NC (*n* = 15 men in each group) days of RT per week for 12 weeks on strength, body composition and RBCs. Muscle strength and body composition (using DXA) were measured before and after 12 weeks of RT. RBC parameters were measured on the first session before RT, and 0 and 24 h after the third RT session in the untrained (week 1 of RT) and trained (week 12 of RT) states. This is the first study to investigate the post-exercise responses of RBC to multiple bouts of RT in untrained and trained states, and how recovery period influences the responses. We hypothesized that: (1) responses would be similar between C and NC groups in all aspects of strength, body composition and RBC parameters, and (2) multiple bouts of RT can induce transient temporal changes in RBC parameters that would be modulated by training.

## Materials and Methods

### Participants

A total of 30 young, healthy and recreationally active men completed this study, which was approved by the Institutional Review Board of Nanyang Technological University. Two other participants (one from each group) withdrew early on in the study due to time constraints. All participants were advised of the purpose of the study and associated risks, and gave written informed consent in accordance with the Declaration of Helsinki prior to the commencement of the study. The lowest statistical power, based on mixed design repeated measures ANOVA, for the effects of interest, which are the interactions involving group and main effects of training and time, is 0.87 with 15 participants in each group as calculated by G^∗^Power version 3.1.9.2 [2 (group) × 2 (training) design; compromise power analysis with medium effect size, *f* = 0.25; beta/alpha ratio of 1; and correlation of 0.5 among repeated measures].

Participants included in the study were 21–35 years old and non-smokers, with a body mass index of < 30 kg⋅m^-2^. Volunteers with poor health were excluded from the study, such as those that (1) failed an exercise stress test; (2) took long term prescribed medications (including traditional Chinese medicine) for heart, blood, lung (except controlled asthma), liver, kidney, or joint conditions; (3) took anabolic steroids or hormones; (4) had consistent readings of systolic blood pressure > 120 mmHg or diastolic blood pressure > 80 mmHg (taken over three readings in first session and repeated on second session if first session had readings that were elevated); or (5) had fasting blood glucose > 6 mmol⋅L^-1^. Volunteers with any other health conditions or injuries that prevented them from performing strenuous physical activity or any RT required by the study were also excluded. Grounds for exclusion in terms of physical activity status were those that (1) did not engage in any regular physical activity at least once a week for 10 min or more; (2) engaged in regular RT for >3 days per week in the last 6 months before the study; (3) engaged in strenuous physical activity for >5 days per week; or (4) competed in powerlifting, weightlifting or bodybuilding. The range of physical activity criteria was to enhance generalization of the study results for physical activity guidelines while excluding competitive/seasoned strength athletes and highly active individuals. To assess participation eligibility, participants completed (1) a questionnaire to assess health status, (2) the Physical Activity Readiness Questionnaire to assess the risks prior to exercise engagement, and (3) the Global Physical Activity Questionnaire to assess habitual physical activity level. Body mass (Mettler-Toledo GmbH ID1 Plus/KCC150s, Albstadt, Germany), height (Seca 242, Hamburg, Germany), blood pressure (Omron HEM-7211, Kyoto, Japan) and fasting blood glucose (LifeScan One Touch Ultra 2, Milpitas, CA, United States) were also measured as part of the screening procedure.

### Experimental Design

**FIGURE 1:**
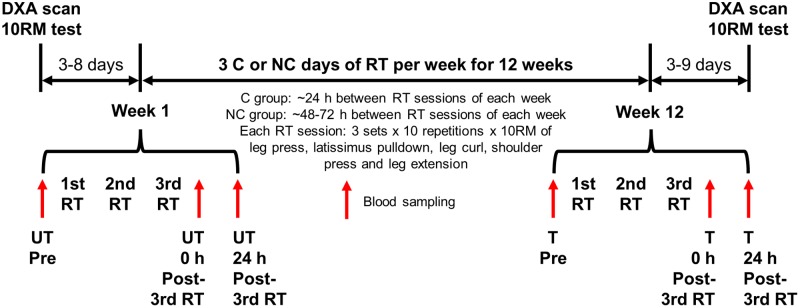
Experimental design. Participants were randomly assigned to either three consecutive (C) or non-consecutive (NC) days of resistance training (RT) per week for 12 weeks, and completed the same procedures except for the recovery period between RT sessions of each week. Blood samples were collected at weeks 1 and 12 of the RT as indicated. DXA, dual-energy X-ray absorptiometry; 10RM, 10-repetition maximum; UT, untrained; T, trained.

Participants were randomly assigned to either 3 C or NC days of RT per week for 12 weeks (**Figure 1**). Groups were balanced for group size (*n* = 15 in each group), and baseline body mass, height, body mass index (BMI) and physical activity level by minimization. Participants in both groups underwent the same procedures including the RT protocol except for the recovery period between RT sessions. RT sessions during each week were separated by ∼24 h of recovery for C group and ∼48–72 h for NC group. C RT sessions were scheduled such that no one performed 6 C days of RT within a 2-week period (e.g., Friday–Wednesday). Outcome measures were 10RM strength, body composition and RBC parameters. Baseline and post-training 10RM and body composition were assessed ∼3–8 days before the first RT session and ∼3–9 days after the last (36th) RT session, respectively. RBC parameters were measured on the first session before RT (Pre), and 0 and 24 h after the third RT session (0 h and 24 h post-3rd RT) in untrained (week 1 of RT) and trained (week 12 of RT) states. To control for diurnal variation, all 10RM assessments and blood draws were conducted in the morning after an overnight fast of 10 h with *ad libitum* water intake, and trained measurements were taken within ∼± 2 h from the time of the day of corresponding untrained measurements. DXA scans were performed in a rested state (and before the 10RM assessment if both were done on the same day) after an overnight fast with *ad libitum* water intake or ≥ 2 h fast following a light meal. For each session with DXA scan, 10RM, or blood sampling, participants wore non-compressive exercise clothing and were required to abstain from (1) any physical activity outside of the study except activities of daily living at least 48 h prior to the session, and (2) alcohol and caffeine at least 24 h prior to the session. Trained baseline blood samples were obtained ∼48 h or more after the previous RT session.

### Dietary Control

Participants were instructed to maintain a consistent diet during the study and required to complete a 2-day dietary record prior to the first and third RT sessions in untrained (week 1) and trained (week 12) states, and ∼ every seven RT sessions in between for a total of 8 × 2-day dietary records. A reference list for portion size estimates and instructions were provided to participants at the start of the study. Dietary intake was monitored over the course of the study and feedback was provided to help participants avoid huge fluctuations in daily caloric intake and macronutrient composition without limiting their caloric intake. Participants were also required to abstain from taking medication (including traditional Chinese medicine) not previously declared unless when ill, hormones, steroids, and supplements (including protein, vitamin and mineral supplements) during the entire duration of the study. Nutritional information of different food/beverage was sourced from various online databases and collated into a common database for use during analyses. The use of a common database helped to reduce inter- and intra-rater discrepancies.

Since caloric intake and macronutrient composition influence some indices of body composition (Aragon et al., 2017), time-varying covariates of daily dietary caloric intake, % fat intake (of daily dietary calories) and % protein intake (of daily dietary calories) were used to adjust for the effects of dietary intake when analyzing body composition changes after 12 weeks of RT. Daily dietary caloric intake was used as a covariate for all body composition parameters except BMC and BMD. In conjunction with daily dietary caloric intake, % fat intake was used to adjust for total and regional fat mass (arm, trunk and leg fat mass), and % body fat; while % protein intake was used to adjust for lean (muscle) mass. Specifically, the average daily dietary caloric intake, % fat intake and % protein intake obtained from the first and second dietary records (i.e., week 1 of RT) were used as covariates for untrained DXA scan measurements, while those from the third to eighth dietary records were used for trained DXA scan measurements.

### Physical Activity Control

Participants were instructed to keep a consistent physical activity level, abstain from any forms of strenuous exercise external to the study and to refrain from commuting by foot or cycle for longer distances, such as to work or school, during the entire study. However, restrictions were not set for short distance walking, such as walking to the bus stop or between classes. Accelerometer (Actigraph wG3TX+, Pensacola, FL, United States) was worn on the right hip at all waking hours throughout the entire study, except when showering or sleeping, to assess physical activity level. ActiLife version 6.9.5 (Actigraph) was used to analyze the data. Tri-axes accelerometer counts were summarized in 1 s epochs and Freedson cut-off points were used to define intensity domains (Sasaki et al., 2011). A valid day was defined as having at least 10 h of daily accelerometer wear time (Choi et al., 2011). Non-wear was defined by intensity of at least 60 consecutive minutes of zero activity counts, with allowance for 1–2 min of counts between 0 and 100 (Troiano et al., 2008). Energy expenditure was calculated based on Freedson VM3 combination (2011) physical activity energy expenditure algorithms (Sasaki et al., 2011). Daily energy expenditure from physical activity was used as a time-varying covariate to adjust for the effects of physical activity on all body composition parameters except BMC and BMD. Specifically, the average daily kcal from physical activity for the first week of RT was used as a covariate for untrained DXA scan measurements, while that from weeks 2 to 12 of the RT was used for trained DXA scan measurements.

### Assessments of 10RM and Body Composition

Participants were familiarized with the equipment and procedures before being assessed for their 10RM in leg press (Technogym Selection, Cesena, Italy), latissimus pulldown (Technogym Pure Strength), leg curl (Technogym Selection), dumbbell shoulder press, and leg extension (Technogym Selection) in the order as listed. This order was used during both 10RM assessments and each RT session. Instead of using a percentage of 1RM for the intensity, 10RM was chosen to ensure that participants maxed out by the 10th repetition. Ten RM corresponds to ∼65–75% 1RM depending on individual variation and this intensity aligns with the recommended guidelines for improving strength and muscle hypertrophy (Garber et al., 2011). For each exercise, the initial load was set relatively light (∼2.5–10 kg) for warm up. If they successfully completed 10 repetitions of the exercise at this load, the weight was increased and they would then attempt another 10 repetitions under the new load. Increments in load (typically 2.5–20 kg) were repeated until participants could only perform 10 repetitions of each exercise and not more. Ten RM was achieved within 5–7 sets including the first warm-up set. Participants were verbally encouraged to give their best effort. During each 10RM testing and RT session, assistance (“spotting”) was given only when participants reached muscular fatigue. DXA (Hologic Discovery W, Marlborough, MA, United States) was used to assess body composition in a rested supine position. Data were analyzed by the same trained personnel.

### RT Protocol

Participants performed three sets of 10 repetitions at their pre-determined 10RM of leg press, latissimus pulldown, leg curl, shoulder press, and leg extension sequentially without warming up. All leg exercises were unilateral but both legs were tested and trained during the entire study. Passive rest of 2 min was given between sets. Each RT session lasted about 45 min. The load for each exercise was increased after every 4–5 weeks if participants could do so in proper form. During each RT session, ingestion of plain water was allowed *ad libitum*. All participants achieved 100% attendance except 1 C and 2 NC participants that missed 1 out of 36 RT sessions. One NC participant had a right knee discomfort during his post-RT 10RM testing for leg curl and thus, the post-RT 10RM for his right leg curl was based on his last week of RT.

### Blood Sampling

Blood sample was taken from an antecubital vein using a 22 G needle and collected in a 3 ml vacutainer with spray-coated K_2_EDTA for a total of six times per participant. Whole blood samples were kept cold (∼4°C) prior to analyses within hours of sample collection. The samples were analyzed by National University Hospital Referral Laboratories, Singapore, Singapore, for RBC count, Hct, Hb concentration, MCV, MCH mass, MCHC, and RDW. Percent change in PV was calculated from measurements of Hct and Hb using the equation derived by Dill and Costill (1974). RBC count at 0 and 24 h post-3rd RT in untrained and trained states were then corrected for PV changes after a bout of RT (Teixeira et al., 2014).

### Statistical Analyses

Data were checked for assumptions prior to application of the appropriate inferential tests. Group differences for participants’ baseline characteristics were assessed using independent *t*-test (for diastolic blood pressure) or Mann–Whitney *U* test (all other characteristics due to non-normality). Ten RM differences were analyzed using 2 (groups: C and NC) × 2 (training: pre- and post-RT) mixed design repeated measures ANOVA with aligned rank transformation for non-normal data with or without equal variances (all exercises except shoulder press). Partial eta squared ($\eta_{p}^{2}$) effect sizes were calculated. Body composition differences were determined using linear mixed models (LMMs) with group and training as fixed categorical variables, and training as a repeated factor. Time-varying covariates of daily dietary caloric intake and daily physical activity energy expenditure (for total, lean and fat mass, and % body fat) plus % fat intake (for total and regional fat mass, and % body fat) or % protein intake (for lean mass) were included to adjust for effects of dietary intake and physical activity. No adjustments were made for BMC and BMD. Differences in RBC parameters and PV were analyzed using LMM with group, training (weeks 1 and 12) and time (pre-exercise, and 0 and 24 h post-3rd RT) as categorical variables, and training and time as repeated factors. For all LMM, different covariance structures were tested and the model with the best fit was selected for each variable. Calculation of effect sizes for LMM is not straightforward. Thus, an equivalent of Cohen’s *d* effect size for pairwise comparison was calculated using the mean difference between the pair divided by the pooled standard deviation, which was calculated from the standard error obtained from the estimated marginal means table. Alpha was set at 0.05 for all tests. Statistical analyses were performed using IBM SPSS version 23. All 95% CI presented in the text represent CI in the unit of the corresponding variable.

## Results

### Participant’s Baseline Characteristics

Both groups had similar age [mean 25 (SD 2) years], height [1.72 (0.06) m], body mass [65 (10) kg], BMI [22.2 (2.7) kg⋅m^-2^], systolic and diastolic blood pressures [114 (5)/69 (8) mmHg], fasting glucose [4.5 (0.3) mmol⋅L^-1^], and physical activity level [2144 (1428) MET-min⋅week^-1^] to begin with (*p* = 0.161–0.999) (**Table 1**). Within each group (*n* = 15 each), three participants did vigorous RT 1–3 days per week while two (C) or three (NC) others did non-vigorous RT using bodyweight (e.g., squats, push-ups, sit ups) or light dumbbells in short duration (≤15–20 min) 2–3 days per week. All, except one NC participant, did moderate or vigorous AT in the form of recreational activities and/or walking/cycling as part of transportation for 4–7 days per week but none exceeded the exclusion criteria of >5 days per week of vigorous physical activity.

**Table 1:** Participants’ baseline characteristics.

			Differences	
Variable^a^	C (*n* = 15)	NC (*n* = 15)	*p*-Value^b^	95% CI^c^
Age (years)	24 (2)	25 (2)	0.595	-2, 1
Height (m)	1.71 (0.07)	1.72 (0.05)	0.539	-0.05, 0.03
Body mass (kg)	65.7 (10.6)	63.8 (9.2)	0.713	-5.9, 8.8
Body mass index (kg⋅m^-2^)	22.6 (2.5)	21.8 (2.9)	0.233	-1.0, 3.0
Systolic blood pressure (mmHg)	116 (5)	113 (5)	0.161	-1, 6
Diastolic blood pressure (mmHg)	70 (8)	67 (8)	0.372	**-3, 8**
Fasting blood glucose (mmol⋅L^-1^)	4.5 (0.3)	4.5 (0.4)	0.744	-0.3, 0.2
Physical activity level (MET-min⋅week^-1^)	2,087 (1,444)	2,201 (1,461)	0.999	-1,000, 840

*^a^Values are presented as means (SD) for descriptive purposes.*

*^b^There were no significant differences between both intervention groups in any of the baseline variables.*

*^c^95% confidence interval (CI) of mean (in bold) or median difference.*

*C, consecutive group; NC, non-consecutive group; MET, metabolic equivalent.*

### Dietary Intake

Dietary intake had a considerable amount of variation within the participants but similar dietary patterns and variability between groups. Therefore, the results are presented as an entire cohort. Mean daily dietary caloric intake was 1,825 kcal⋅d^-1^ with a mean CV of 22% within participants and mean range of 1,453 kcal⋅d^-1^ within participants (i.e., mean difference between minimum and maximum daily caloric intake within each participant is 1,453 kcal). Mean % fat intake was 32% with mean CV of 22% and mean range of 24% within participants. Mean % protein intake was 18% with mean CV of 23% and mean range of 14% within participants. Mean % carbohydrate intake was 51% with mean CV of 15% and mean range of 27% within participants. Such within-participant variations highlight the need to monitor dietary intake at multiple time points during the study and use time-varying (instead of time-invariant) covariates, of which both were done in this study even though the data were reduced to two time-points for the covariates (pre- and post-RT).

### Physical Activity

Both groups complied well to the physical activity restrictions. C group had a mean (SD) wear time compliance of 74 (20)% (min–max: 40%–99%) out of 84 days. Daily caloric expenditure from physical activity was 598 (134) kcal⋅day^-1^ (min-max: 403–799 kcal⋅day^-1^) and only 13 (3)% (min–max: 9%–17%) of valid wear time (i.e., ≥ 10 h of waking hours per day including RT days) was spent on MVPA (% MVPA). Physical activity level within participants was consistent; mean CV of % MVPA within participants was 9 (5)% (min–max: 4%–19%). NC group had an 89 (13)% (min–max: 49%–100%) wear time compliance. Daily caloric expenditure was 600 (105) kcal⋅day^-1^ (min–max: 409–830 kcal⋅day^-1^) and % MVPA was 13 (2)% (min–max: 10%–17%). Mean CV of % MVPA within participants was 7 (2)% (min–max: 3%–9%).

### Strength Changes

**FIGURE 2:**
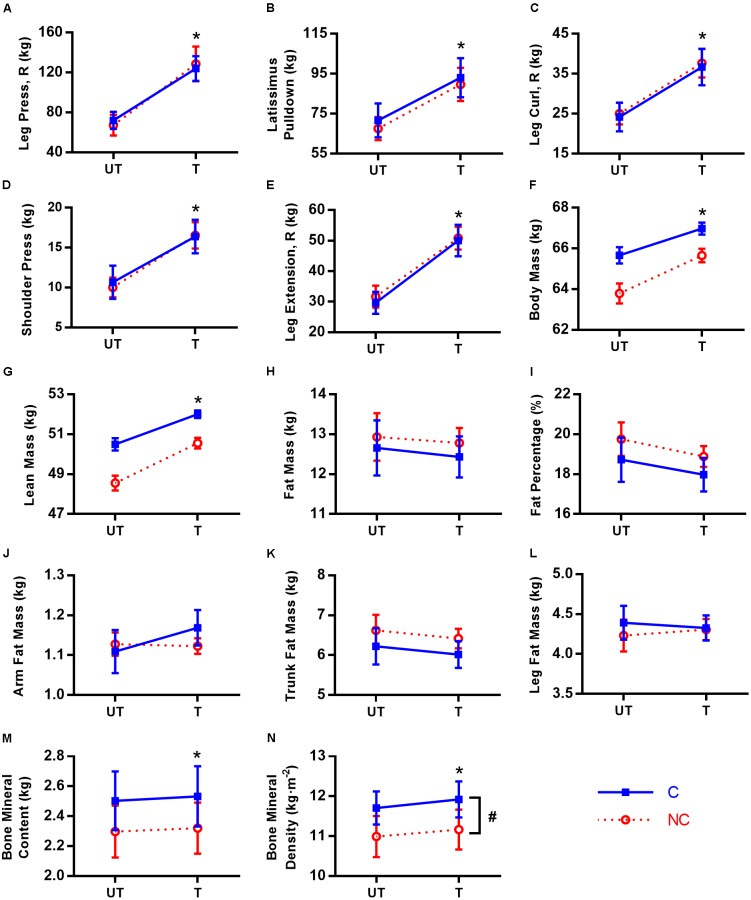
Ten-repetition maximum and body composition in untrained (UT) and trained (T) states for consecutive (C, 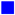) and non-consecutive (NC, 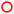) groups for: leg press with right leg **(A)**, latissimus pulldown **(B)**, leg curl with right leg **(C)**, shoulder press **(D)**, leg extension with right leg **(E)**, body mass **(F)**, lean mass **(G)**, fat mass **(H)**, fat percentage **(I)**, arm fat mass **(J)**, trunk fat mass **(K)**, leg fat mass **(L)**, bone mineral content **(M)**, and bone mineral density **(N)**. Values are means with 95% confidence interval. For **(F–L)**, means are calculated from predicted values after adjustment for covariates. No significant training × group interaction for all parameters (*p* = 0.075–0.974). ^∗^Significant difference between UT and T states (*p* < 0.001). ^#^Significant group difference between C and NC (*p* = 0.025).

No significant training × group interaction and group effect was found for all strength parameters (*p* = 0.075–0.974; $\eta_{p}^{2}$ < 0.01–0.11, small effect sizes), and the parallel lines are strong evidence for the absence of interaction (**Figure 2**). The full set of results is presented in **Table 2**. Only main effect of training was significant for all strength parameters (*p* < 0.001; $\eta_{p}^{2}$ = 0.79–0.92, large effect sizes), meaning both groups increased in strength to a similar extent following 12 weeks of RT for all exercises. The *a priori* power for a 2 × 2 mixed design (in this context) is the same for both interaction and within-subject main effects. The fact that significant and large training effect was found for all parameters is another strong indicator that the lack of differential responses between groups (i.e., no interaction) is not because the study was underpowered.

**Table 2:** Changes in ten-repetition maximum (10RM) strength and body composition.

	C (*n* = 15)		NC (*n* = 15)		*p*-Value^b^ (effect size)^c^		
Variable^a^	Untrained	Trained	Untrained	Trained	Training	Group	Interaction
**10RM strength**							
Leg press, L (kg)	70 (17)	121 (24)	64 (18)	120 (29)	**<0.001** (0.88)	0.242 (0.05)	0.781 (<0.01)
Leg press, R (kg)	72 (16)	124 (23)	67 (19)	129 (31)	**<0.001** (0.87)	0.431 (0.02)	0.505 (0.02)
Latissimus pull (kg)	72 (15)	93 (18)	68 (10)	90 (15)	**<0.001** (0.79)	0.360 (0.03)	0.837 (<0.01)
Leg curl, L (kg)	24 (6)	37 (8)	25 (5)	37 (7)	**<0.001** (0.92)	0.782 (< 0.01)	0.710 (< 0.01)
Leg curl, R (kg)	24 (7)	37 (8)	25 (5)	38 (7)	**<0.001** (0.91)	0.653 (<0.01)	0.974 (<0.01)
Shoulder press (kg)	11 (4)	16 (4)	10 (2)	17 (3)	**<0.001** (0.90)	0.815 (<0.01)	0.304 (0.04)
Leg extension, L (kg)	29 (6)	49 (8)	30 (5)	50 (7)	**<0.001** (0.91)	0.552 (0.01)	0.122 (0.08)
Leg extension, R (kg)	30 (7)	50 (9)	32 (7)	51 (7)	**<0.001** (0.91)	0.930 (<0.01)	0.075 (0.11)
**Body composition**							
Body mass (kg)	65.7 (10.6)	67.0 (10.4)	63.8 (9.2)	65.7 (9.5)	UA: **<0.001** A: **<0.001** (0.17)	0.662 0.589 (0.20)	0.347 0.140
Lean mass (kg)	50.5 (6.5)	52.0 (5.9)	48.6 (5.4)	50.6 (5.5)	UA: **<0.001** A: **<0.001** (0.29)	0.428 0.451 (0.28)	0.405 0.825
Fat mass (kg)	12.7 (5.6)	12.4 (5.6)	12.9 (5.3)	12.8 (5.7)	UA: 0.438 A: 0.994 (<0.001)	0.878 0.991 (0.004)	0.885 0.305
Fat percentage (%)	18.7 (6.4)	18.0 (6.1)	19.8 (6.0)	18.9 (6.4)	UA: **0.029** A: 0.218 (0.09)	0.668 0.803 (0.09)	0.866 0.475
Arm fat mass (kg)	1.11 (0.56)	1.17 (0.61)	1.13 (0.54)	1.12 (0.57)	UA: 0.317 A: 0.154 (0.08)	0.948 0.908 (0.04)	0.242 0.472
Trunk fat mass (kg)	6.22 (2.83)	6.02 (2.83)	6.62 (2.96)	6.42 (3.13)	UA: 0.172 A: 0.622 (0.03)	0.709 0.860 (0.07)	0.996 0.305
Leg fat mass (kg)	4.39 (2.27)	4.33 (2.24)	4.23 (2.13)	4.31 (2.31)	UA: 0.964 A: 0.586 (0.03)	0.913 0.806 (0.09)	0.437 0.156
Bone mineral content (kg)	2.504 (0.356)	2.533 (0.364)	2.297 (0.313)	2.320 (0.310)	UA: **<0.001** (0.08) A: NA	0.099 (0.62) NA	0.628 NA
Bone mineral density (kg⋅m^-2^)	11.71 (0.75)	11.92 (0.81)	10.99 (0.94)	11.17 (0.90)	UA: **<0.001** (0.23) A: NA	**0.025** (0.87) NA	0.632 NA

*^a^Values are presented as means (SD) for descriptive purposes.*

*^b^Bold text indicates statistical significance (p ≤ 0.05).*

*^c^Effect size using partial eta squared for 10RM strength or Cohen’s d for body composition (adjusted for covariates if applicable).*

*C, consecutive group; NC, non-consecutive group; L, left leg; R, right leg; UA, unadjusted for covariates; A, adjusted for covariates; NA, not applicable as measurements were not adjusted.*

For leg press, the mean 10RM increase in both legs was 55 kg. The 10RM for left leg press increased 80% from 67 to 121 kg (95% CI [45, 60], $\eta_{p}^{2}$ = 0.88), while that for right leg increased 81% from 70 to 126 kg (95% CI [50, 65], $\eta_{p}^{2}$= 0.87). The 10RM for latissimus pulldown increased 31% from 70 to 91 kg (95% CI [18, 25], $\eta_{p}^{2}$ = 0.79). For leg curl, the mean 10RM increase in both legs was 13 kg. The 10RM for left leg curl increased 53% from 24 to 37 kg (95% CI [11, 14], $\eta_{p}^{2}$= 0.92), while that for right leg increased 51% from 25 to 37 kg (95% CI [11, 14], $\eta_{p}^{2}$ = 0.91). The 10RM for shoulder press increased 59% from 10 to 16 kg (95% CI [5, 7], $\eta_{p}^{2}$ = 0.90). For leg extension, the mean 10RM increase in both legs was 20 kg. The 10RM for left leg extension increased 68% from 30 to 50 kg (95% CI [18, 23], $\eta_{p}^{2}$ = 0.91), while that for right leg increased 64% from 31 to 50 kg (95% CI [18, 21], $\eta_{p}^{2}$ = 0.91).

### Body Composition Changes

The predicted values adjusted for covariates for body composition parameters (no covariate adjustment for BMC and BMD) are presented in **Figure 2**. Similar to strength changes, no significant interaction was found for all body composition parameters (*p*= 0.140–0.825), with the parallel lines in most parameters as strong evidence. Even prior to adjustment for covariates, there was no interaction for all parameters as well (*p*= 0.242–0.996, **Table 2**). Similarly, the conclusions for main effects of training and group are also the same between adjusted and non-adjusted values for all parameters except % body fat (elaborated below) and hence, only adjusted results will be highlighted unless otherwise stated. We found significant training and group (lowest *a priori* power) differences, again demonstrating that the study had adequate power to detect interactions.

Both groups responded similarly in body composition post-RT (**Table 2**). RT increased total body mass by 1.7 kg (95% CI [1.1, 2.3], *p* < 0.001, *d* = 0.17) via increases in lean mass by 1.6 kg (95% CI [1.0, 2.3], *p*< 0.001, *d* = 0.29) and BMC by 26 g, (95% CI [14, 38], *p* < 0.001, *d* = 0.08). Although C group has higher BMD than NC (0.73 kg⋅m^-2^ denser; 95% CI [0.10, 1.37], *p* = 0.025, *d* = 0.87), both groups also increased their BMD similarly following RT (average increase of 0.19 kg⋅m^-2^, 95% CI [0.11, 0.28], *p* < 0.001, *d* = 0.23). There were no other group differences [*p* = 0.099 (for BMC)–0.991] and RT did not change total and regional (arm, trunk, and leg) fat mass (*p* = 0.154–0.994), and % body fat (-0.5% points, 95% CI [-1.3, 0.3], *p* = 0.218, *d* = 0.09). However, unadjusted % body fat was reduced post-RT (-0.8% points, 95% CI [-1.5, -0.1], *p* = 0.029, *d* = 0.13).

### PV and RBC Changes

**FIGURE 3:**
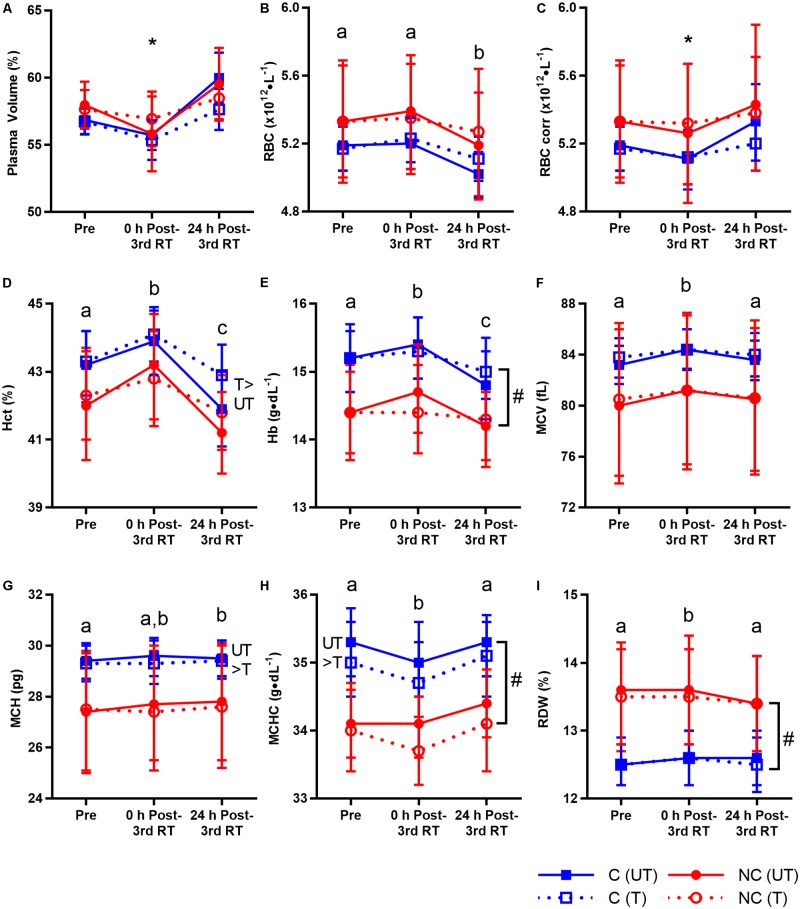
Plasma volume and red blood cell (RBC) parameters before resistance training (RT) at first session (Pre), and 0 and 24 h post-3rd RT session in untrained (UT, solid lines) and trained (T, dotted lines) states for consecutive (C, blue squares) and non-consecutive (NC, red circles) groups: plasma volume **(A)**, RBC count **(B)**, RBC count corrected for plasma volume changes **(C)**, hematocrit **(D)**, hemoglobin **(E)**, mean corpuscular volume **(F)**, mean corpuscular hemoglobin **(G)**, mean corpuscular hemoglobin concentration **(H)**, and RBC distribution width **(I)**. Values are means with 95% confidence interval. No significant interaction for all parameters (*p*= 0.076–0.994) except corrected RBC count (^∗^significant time × training interaction, *p* = 0.001; details in text). Significant main effect of time for all parameters (*p* < 0.001–0.003). Different letters indicate significant difference from other time points (*p* < 0.001–0.040). Comparison between UT and T states indicates significant difference between them (*p* = 0.001–0.041). ^#^Significant group difference between C and NC (*p* = 0.006–0.030).

For better evaluation of hematological changes post-exercise, RBC count were corrected for PV changes (Teixeira et al., 2014) at 0 and 24 h post-3rd RT in untrained and trained states. Time × training interaction was significant (*p* = 0.014) with no other significant interaction (*p* = 0.314–0.630) for PV (**Figure 3A**). In the untrained state, there was a trend for PV to decrease at 0 h post-3rd RT (-2.8% from 57.5 to 55.9%, *p* = 0.067) before increasing significantly at 24 h post-3rd RT compared to Pre (4.1%, 95% CI [0.3, 4.4], *p* = 0.021, *d* = 0.71) and 0 h post-3rd RT (7.0% from 55.9 to 59.8%, 95% CI [2.6, 5.3], *p* < 0.001, *d* = 1.04). Following 12 weeks of RT, there was no change in baseline PV (57.5% versus 57.2%; *p*= 0.336) and the pattern of responses is similar but more suppressed. Only an increase of 3.5% from 56.2% at 0 h post-3rd RT to 58.2% at 24 h post-3rd RT (95% CI [0.7, 3.2]) was significant (*p* = 0.001, *d* = 0.67).

There was no interaction of any kind for all RBC parameters (*p* = 0.076–0.994) except a time × training interaction (*p* = 0.001) for corrected RBC count (**Figure 3**). There were time, training and group differences, again demonstrating that the study had adequate power to detect group interactions. Exercise decreased corrected RBC count at 0 h post-3rd RT compared to Pre in the untrained state (-1.5%, 95% CI [-0.15, -0.01], *p* = 0.021, *d* = 0.15) and 24 h post-3rd RT in both untrained (-3.7%, 95% CI [-0.27, -0.12], *p* < 0.001, *d* = 0.32) and trained states (-1.4%, 95% CI [-0.12, -0.02], *p* = 0.003, *d* = 0.15).

Temporal changes were significant for all RBC parameters. Uncorrected RBC count decreased at 24 h post-3rd RT compared to Pre (-1.9%, 95% CI [-0.16, -0.04], *p* < 0.001, *d* = 0.22) and 0 h post-3rd RT (-2.7%, 95% CI [-0.20, -0.08], *p* < 0.001, *d* = 0.31). Hct increased 1.7% 0 h post-3rd RT compared to Pre (95% CI [0.3, 1.2], *d* = 0.37) before decreasing 3.4% at 24 h post-3rd RT (95% CI [-1.9, -1.0], *d* = 0.75) and falling 1.7% below Pre level (95% CI [-1.2, -0.3], *d* = 0.37), all *p* < 0.001. Similar to Hct, Hb increased 1.0% 0 h post-3rd RT compared to Pre (95% CI [0.0, 0.3], *p* = 0.040, *d* = 0.16) before decreasing 2.5% at 24 h post-3rd RT (95% CI [-0.5, -0.2], *p* < 0.001, *d* = 0.40) and falling 1.5% below Pre level (95% CI [-0.4, -0.1], *p* = 0.005, *d* = 0.24).

Morphological properties of RBC were also altered post-exercise. MCV increased at 0 h post-3rd RT compared to Pre (1.0%, 95% CI [0.5, 1.1], *p* < 0.001, *d* = 0.11) and 24 h post-3rd RT (0.7%, 95% CI [0.3, 0.9], *p* < 0.001, *d* = 0.08). MCH increased 0.5% 24 h post-3rd RT compared to Pre (95% CI [0.0, 0.2], *p* = 0.005, *d* = 0.04). MCHC decreased at 0 h post-3rd RT compared to Pre (-0.7%, 95% CI [-0.4, 0.0], *p* = 0.011, *d* = 0.27) and 24 h post-3rd RT (-1.0%, 95% CI [-0.5, -0.1], *p* < 0.001, *d* = 0.36). Similar to MCV, RDW increased at 0 h post-3rd RT compared to Pre (0.6%, 95% CI [0.0, 0.2], *p* = 0.011, *d* = 0.08) and 24 h post-3rd RT (0.8%, 95% CI [0.0, 0.2], *p* < 0.001, *d* = 0.09).

While RT increased Hct by 0.8% (95% CI [0.0, 0.7], *p* = 0.026, *d* = 0.18), RT decreased MCH (-0.4%, 95% CI [-0.2, 0.0], *p* = 0.041, *d* = 0.04) and MCHC (-0.7%, 95% CI [-0.4, -0.1], *p* = 0.001, *d* = 0.29). C group on average has higher Hb and MCHC than NC group (Hb: 15.2 compared to 14.4 g/dL, 95% CI [0.1, 1.4], *p* = 0.030, *d* = 0.83; MCHC: 2.8% higher, 95% CI [0.3, 1.6], *p* = 0.006, *d* = 1.08). RDW was 6.7% lower in C group compared to NC group (95% CI [-1.7, -0.1], *p* = 0.023, *d* = 0.86). There were no other training (*p* = 0.178–0.797) and group differences (*p* = 0.130–0.422).

Mean values of all RBC parameters (including RBC corrected for PV changes) for both groups were within normal clinical range at baseline and post-exercise regardless of training status (**Supplementary Table S1**). However, RBC (uncorrected and corrected), Hb and MCHC had 1–4 participants in C or NC group with normal baseline values but RT-induced out of range values at 0 and/or 24 h post-3rd RT in the corresponding untrained or trained states (maximum 4 out of 30 participants at any time point per parameter; data not shown). Such occurrences were similar between C (*n* = 5) and NC (*n* = 4) groups collectively across these three parameters.

## Discussion

This study aimed to determine the effects of 3 C or NC days of RT per week for 12 weeks on strength, body composition and RBCs. The results revealed no interaction involving group in all measures of strength, body composition, and RBC parameters as hypothesized. Both C and NC RT are equally effective in improving strength, lean mass, BMC and BMD that are comparable to those reported by other studies (elaborated below). There is no change in fat mass or fat distribution for both groups post-RT. This is the first study to investigate responses of RBC parameters after multiple bouts of RT in untrained and trained states, and whether recovery period modulates the responses. RBC responses did not differ between groups. There was no interaction and RT induced temporal changes in all RBC parameters except RBC corrected for PV changes (time × training interaction). A novel finding is that all RBC parameters returned to baseline at 24 h post-3rd RT regardless of training status except Hct and Hb that were influenced by PV changes, and MCH that was partly affected by Hb (elaborated below). Chronic RT increased Hct and lowered MCH and MCHC, but did not alter uncorrected RBC, Hb, MCV and RDW. Regardless of the changes, mean values of all RBC parameters were still within normal range. However, for a small number of participants who had normal baseline values of RBC parameters, strenuous RT induced out of range values in RBC (uncorrected and/or corrected), Hb and/or MCHC even at 24 h post-3rd RT in untrained or trained states. Therefore, it is best to avoid strenuous physical activity for at least 48 h prior to a full blood count measurement to avoid false positive readings.

### Strength and Body Composition Changes

One critical observation from this study is that despite not adhering to the recommended 48–72 h of recovery between RT sessions, the C group did not exhibit attenuated increments in strength, lean mass, BMC or BMD when compared to the NC group. Our findings agree with those of the only study that directly compared C and NC days of RT. Carvalho and Rodrigues Santos (2016) found similar improvements in 1RM bench press and leg press, and body composition between both groups after 7 weeks of 3 days per week of RT. Collectively, these results are not baseless. Although some acute animal (Haddad and Adams, 2002) and human (Bickel et al., 2005; Flores et al., 2011; Radaelli et al., 2012; Korak et al., 2015) studies do point to at least 48 h of recovery between RT sessions for better strength recovery and muscle growth, other chronic RT studies are inconclusive regarding the optimal recovery period and do not indicate that C RT is indeed inferior to NC RT. Hunter (1985) found 4 C days to be superior to 3 NC days of RT per week, despite the same weekly volume, in increasing maximum bench press and bench press endurance after 7 weeks. While the better results with C RT was attributed to the higher frequency, one could also attribute it to C RT being superior to NC RT. In another study, Gillam (1981) had participants perform 9 weeks of intense RT of 1, 2, 3, 4, or 5 C days per week and the group with 5 C days per week had the best strength improvement. Each day of RT was the same and thus, while one could argue that 5 C days per week had the best outcome because of the higher volume and/or frequency, the result certainly does not support the notion that strength adaptation is compromised with C RT.

Improvements of 31–81% amounting to large $\eta_{p}^{2}$ of 0.79–0.92 in 10RM strength observed in this study are similar to or greater than those reported in other studies [12–52% over 3–6 months (reviewed by Ralston et al., 2017) or 12–21% over 6 weeks (Paulsen et al., 2003)] of RT with similar weekly volume and intensity (3 sets × 7–12 repetitions × 7RM–12RM × 3 days per week) in young untrained or trained males for the similar exercises. Such increments seen in this study reflect the relatively untrained status of our participants (for RT) on average at baseline, and the robust RT stimulus provided in this study. The latter adds credibility to our study as it meant that the lack of differential responses between C and NC groups was not due to an ineffective RT program. In fact, every participant improved their 10RM in all five exercises except 1 C participant in the latissimus pulldown and another 1 C participant in the shoulder press. Both were the second strongest out of both groups for the respective exercise that they did not improve. C RT *per se* did not impair the ability to improve strength because the strongest participants in latissimus pulldown and shoulder press were also in the C group and did improve their strength further.

Two other noteworthy findings from our study were that recovery requirement did not differ between (1) upper and lower body exercises, and (2) single- and multi-joint exercises, contrary to popular beliefs, since both groups improved 10RM similarly in all five exercises. These results echoed the findings of Korak et al. (2015) and Carvalho and Rodrigues Santos (2016), with one exception. Korak et al. (2015) found that multi-joint exercises required longer recovery period compared to single-joint exercises, and that bench press and deadlift required longer recovery period of more than 48 h. The finding on bench press contradicted that by Carvalho and Rodrigues Santos (2016) who found no difference in 1RM bench press between C and NC groups after 7 weeks of RT. This discrepancy could be due to one being an acute study (Korak et al., 2015) and the other a long-term study (Carvalho and Rodrigues Santos, 2016). Deadlift was not performed in this study and that by Carvalho and Rodrigues Santos (2016). Future chronic studies with an array of exercises are needed to explore if differential recovery between multi- and single-joint exercises exist.

RT-induced muscle growth is well established and the 1.6 kg (3.3%) lean mass gain observed in this study is consistent with the -0.5 kg to 1.5 kg changes observed in young participants of varying training status as reviewed by Morton et al. (2017), and the 2 kg (3.4%) increase in young, inactive men post-RT (McCarthy et al., 1997). These comparable or superior improvements in strength for all exercises and lean mass demonstrated the robust stimuli provided by the RT in our study. The RT program is concordant with recommendations by ACSM except the recovery period for the C group (Garber et al., 2011). We kept the sets, repetitions and relative intensity constant while adjusting the load as this is a common RT protocol reported in the literature (Ralston et al., 2017; Schoenfeld et al., 2017), especially for novice or intermediate exercisers to RT. Carvalho and Rodrigues Santos (2016) and McCarthy et al. (1997) used an undulating periodization approach in which each of the three RT sessions of the week was different in intensity and volume, and also found similar improvements in strength and body composition between C and NC groups, and comparable lean mass gain to our results, respectively.

In the present study, participants increased total body BMC by 1.1% (26 g) and BMD by 1.7%. The inclusion of bone results from this study is valuable because RT studies on BMD in young males are limited as most intervention studies focused on women and older adults due to the high prevalence of osteoporosis in these populations. McCarthy et al. (1997) found a similar, albeit non-significant, increase of 0.6% (16 g) in young, inactive men after 12 weeks of RT. Young, recreationally active males increased BMD at the lumbar spine by 7.7% and femoral neck by 4.2% after 24 weeks of RT (Almstedt et al., 2011). Male college rowers increased 2.9% in BMC and 4.2% in lumbar spine BMD after a 7-month training program of 8 h rowing, 1 h RT and 1 h running per week (Cohen et al., 1995).

No significant change in fat mass (+2 g adjusted for covariates, 95% CI [-533, 537], *p*= 0.994, *d* < 0.001), fat distribution and % body fat (-0.5% points adjusted for covariates) was found in both groups. Our participants were on average non-overweight (**Table 1**). Results from this study add to the limited pool of data on fat loss with RT in young, healthy, non-obese individuals, and agree with the common findings that RT alone is ineffective for reduction in fat mass and % body fat in this population (Poehlman et al., 2000; Lemmer et al., 2001; Olson et al., 2007). However, McCarthy et al. (1997) did find a large 1.9 kg (12.0%) decrease in fat mass and 2.7% points reduction in % body fat from 20.4% in young, healthy, normal weight (BMI 25.1 kg⋅m^-2^) men using an undulating periodized RT program. Given the paucity of comparisons in different RT periodization approaches to fat loss, it remains to be seen if this is a major contributor to the discrepancy. On the other hand, diet is a potent cofounding factor for body composition, but there is limited information on dietary intake and control in these studies.

### PV Changes

Acute and chronic PV responses did not differ between the two groups. Instead, RT-induced transient post-exercise changes were modulated by training status (time × training interaction). While there was a pattern of small, non-significant decrease of PV immediately post-3rd RT (-2.8% and -1.7% in untrained and trained states, respectively), our results contrasted with the decrease in PV commonly observed immediately after a bout of RT, ranging from -6.9 to -10.4% for intensity of 8RM–12RM or 70–80% 1RM (Wallace et al., 1990; Ahmadizad and El-Sayed, 2005; Ahmadizad et al., 2006; Cakir-Atabek et al., 2009; Teixeira et al., 2014). Such decreases were still evident when hydration status was controlled for (-22.6%, Craig et al., 2008) or sweat loss during the RT session was replaced with fluid intake (-7.5%, Ahmadizad et al., 2006). A more plausible explanation is the difference in posture during blood sampling and RT. Just by changing from a supine position to a leg press posture, there was a PV drop of 10–15% across the three times that this condition was repeated (Craig et al., 2008). When 3 sets × 10 repetitions × 10RM leg presses were performed, PV dropped a further of ∼10% points. In our study, all were seated/standing exercises but all blood samples were taken in a supine position. It is likely that by resuming a supine position, PV may increase by 10–15% and thereby negating any RT-induced PV drop to non-significant amount. We assumed a supine position for blood sampling for ethics approval as a preventive measure should participants get lighted headed during the procedure.

Less is known about PV changes after 24 h post-RT. Wallace et al. (1990) reported a 7% PV expansion 24 h after 7 sets × 8–12 repetitions × 8RM–12RM for seven exercises in recreational lifers while we observed PV expansion of 4.1% and 1.7% (non-significant) 24 h post-3rd RT in the untrained and trained states, respectively. The subdued responses in our study could be due to a lower volume (3 sets × 5 exercises). Similarly, research on chronic effects of RT on PV is sparse but our finding of non-significant change in resting PV (-0.5%) after 12 weeks of RT is consistent with those of others that found changes of -0.4% to -1.8% (Cakir-Atabek et al., 2009; Hulmi et al., 2010) or +2.2% (non-significant, McCarthy et al., 1997). However, suppression of post-exercise PV changes with chronic RT in our study (**Figure 3A**, gentler slopes in trained states) was not observed by Cakir-Atabek et al. (2009) (-6.85% and -8.07% 0 h post-RT in weeks 1 and 6, respectively). Interestingly, Kilic-Toprak et al. (2012) found increases (significance not tested) of 3.8%, 2.4%, and 2.9% 0 h post-RT in weeks 1, 4, and 12 but the different intensity and volume used in weeks 1, 2, 3, and 4–12 made the interpretation challenging. More studies are needed to investigate whether chronic RT influences resting values and post-exercise responses of PV.

### RBC Changes

Echoing our findings on strength and body composition, this is the first study to show that recovery period of 24–72 h does not affect the acute and chronic responses to RT differently in PV and all RBC parameters. Without similar studies for comparisons, we focus on the temporal and training changes to corroborate our findings. Given the effect of PV on RBC count, we compared post-exercise RBC count corrected for PV changes instead of uncorrected values. If corrected values were not provided, we calculated them from the PV changes reported (Ahmadizad et al., 2006; Cakir-Atabek et al., 2009) or estimated from Hb and Hct (Kilic-Toprak et al., 2012) using the equation by Dill and Costill (1974), and indicate “significance not tested.” Like PV, RT-induced temporal changes in corrected RBC count were modulated by training. Most studies (Ahmadizad and El-Sayed, 2005; Ahmadizad et al., 2006; Cakir-Atabek et al., 2009; Teixeira et al., 2014) had no change or a decrease ranging from -2.6 to -4.1% right after a bout of RT in agreement with the -1.5% change (untrained state) 0 h post-3rd RT in our study. However, Kilic-Toprak et al. (2012) saw 0 h post-RT increases (significance not tested) of 3.5% at week 1 and 2.8% at week 4. The acute decreases could be due to increased hemolysis as observed with 6 weeks of RT (Schobersberger et al., 1990) as a result of mechanical stress from large muscular contractions (Mairbäurl, 2013). Future acute RT studies should be conducted to investigate this further. The magnitude of decrease seems to be influenced by intensity and repetitions with no change after 3 sets × 6 repetitions × 85% 1RM but -3.2% (significance not tested) after 3 sets × 12 repetitions × 70% 1RM (Cakir-Atabek et al., 2009).

To our knowledge, no RT study has investigated the 24 h post-exercise responses for RBC parameters but PV and RBC returned to basal levels 30 min after a bout of RT (Ahmadizad and El-Sayed, 2005; Ahmadizad et al., 2006; Teixeira et al., 2014), similar to our finding of no change in corrected RBC count at 24 h post-3rd RT regardless of training status. Taken together, it is likely that corrected RBC count returned to basal level by 24 h after a bout of RT and thus, recovery period of 24–72 h did not matter and both groups had similar responses 24 h post-3rd RT. Following chronic RT of 18–42 sessions, most studies reported no change in resting RBC volume (Hulmi et al., 2010) or count (Schobersberger et al., 1990; Hu et al., 2008; Cakir-Atabek et al., 2009; Kilic-Toprak et al., 2012) like what we found. However, increasing the length of RT from 10 to 20 weeks (frequency not specified; Hu et al., 2008) and/or using an undulating periodization approach increased basal RBC volume (McCarthy et al., 1997) or count (Kilgore et al., 2002; Hu et al., 2008). Chronic RT seemed to suppress post-exercise responses in corrected RBC count [**Figure 3C** and the study by Kilic-Toprak et al. (2012): 3.5%, 2.8%, and 2.4% (significance not tested) 0 h post-RT in weeks 1, 4, and 12, respectively]. Furthermore, no change in corrected RBC count 0 h post-RT was observed in trained bodybuilders (Teixeira et al., 2014). Similar to PV, such suppression in post-exercise responses with chronic RT in corrected RBC count was not observed by Cakir-Atabek et al. (2009); -3.2% in week 1, -3.6% in week 6 for 70% 1RM, and 0.0% in week 1, -0.8% in week 6 for 85% 1RM].

For all other RBC parameters, there was no interaction but RT-induced temporal changes. While there is a confusing array of changes from decrease, no change, to increase in Hct and Hb based on statistical significance, the pattern of change in them is clear and consistent considering the absolute effect sizes in conjunction with PV changes. Hct at 0 h post-RT increased with decreased PV (Ahmadizad and El-Sayed, 2005; Ahmadizad et al., 2006; Craig et al., 2008; Cakir-Atabek et al., 2009; Teixeira et al., 2014), or decreased with increased PV (Kilic-Toprak et al., 2012). All these authors attributed the acute changes in Hct and Hb mostly to PV changes. Hb mimicked changes in Hct in direction and magnitude [within 0.8% points; (Ahmadizad and El-Sayed, 2005; Ahmadizad et al., 2006; Craig et al., 2008; Cakir-Atabek et al., 2009); Hb not measured (Teixeira et al., 2014)] except Hct increased 0.6% but Hb decreased 2.8% 0 h post-RT at week 4, and a 2.2% point difference at week 1 in the study by Kilic-Toprak et al. (2012). Hb changes generally mirror those of Hct because the total amount of Hb is dependent on Hct and the amount of Hb in each cell, of which the latter is generally saturated and constant in healthy individuals (Austin et al., 2011). Likewise, our results for Hct and Hb at both time points had similar direction and magnitude of change (0.2–0.9% points difference) and were inversely related to PV. Thus, Hct and Hb dipped below baseline 24 h post-3rd RT due to PV expansion (based on absolute change). In all, results from this study and others (Ahmadizad and El-Sayed, 2005; Ahmadizad et al., 2006; Cakir-Atabek et al., 2009) suggest that any transient RT-induced hemolysis is of a smaller influence than PV decrease, resulting in a net increase in Hct and Hb 0 h after one or multiple bouts of RT.

Transient 0 h post-3rd RT increases in MCV (1%) and RDW (0.6%), and decrease (-0.7%) in MCHC as observed in our study, indicate a higher number of young erythrocytes. Young RBCs are usually larger, leading to a rise in RDW and MCV, and drop in MCHC. Furthermore, young RBCs are characterized by improved deformability (Mairbäurl, 2013). Increased RBC deformability has been observed immediately after a bout of RT in untrained (Cakir-Atabek et al., 2009) or trained state (Kilic-Toprak et al., 2012), and has been associated with increased MCV and decreased MCHC (von Tempelhoff et al., 2016). These results suggest that RT acutely accelerates the turnover of RBCs with increased hemolysis while increasing erythropoiesis to maintain hemostasis. Along with the non-significant change in MCH (0.2%), our findings agree with those of other RT studies: -0.2% (non-significant) to 0.6% in MCV; -0.2% to 0.9% in MCH (all calculated values as MCH is seldom reported); and -3.5% to 0.6% (both limits non-significant) in MCHC immediately after a bout of RT (Ahmadizad and El-Sayed, 2005; Ahmadizad et al., 2006; Cakir-Atabek et al., 2009; Kilic-Toprak et al., 2012). The exception is a significant 0.4% decrease in MCV 0 h post-RT at week 12 and larger changes in MCH of -2.7%, -3.1%, and -1.0% at weeks 1, 4, and 12, respectively, in the study by Kilic-Toprak et al. (2012). Acute or chronic effects of RT on RDW are seldom reported. One study found non-significant change of -11.2% in RDW immediately after a bout of 35% 1RM RT (Ghanbari-Niaki and Tayebi, 2013) and the discrepancy could be due to differences in RT protocol. All RBC indices returned to baseline by 24 h post-3rd RT except for a small increase of 0.5% in MCH (**Figure 3**), due to a slightly more gradual decline (i.e., slower kinetics) in Hb (-2.5%) than RBC (-2.7%) from 0 to 24 h post-3rd RT. MCH is 10× the ratio of Hb to RBC.

Following chronic RT, Hct increased 0.8% on average (main effect of training with no interaction) compared to untrained state with non-significant change of 0.1% in Hb. Only two other studies investigated both acute and chronic effects of RT on RBC parameters but the authors did not directly test the effect of training (Cakir-Atabek et al., 2009; Kilic-Toprak et al., 2012). Therefore, we compared all our results of training effect with the resting values of corresponding parameters reported by others. Our results agree with the direction of change in resting values following chronic RT as reported by others [Hct: 0.4–3.2% and Hb: 0.6–2.2% (Kilgore et al., 2002; Cakir-Atabek et al., 2009; Hulmi et al., 2010; Kilic-Toprak et al., 2012), or not significantly different from controls at week 10 (Hu et al., 2008)]. This range of varying changes in Hct and Hb may be partly due to seasonal variation (Kristal-Boneh et al., 1997; Hu et al., 2008), along with seasonal variation in PV (Kristal-Boneh et al., 1997). An important note, our study was conducted in Singapore, which does not have four seasons. We observed lower MCH (-0.4%) and MCHC (-0.7%) with non-significant changes in MCV (0.3%) and RDW (-0.7%) following RT. Others (Hu et al., 2008; Cakir-Atabek et al., 2009) found non-significant changes in MCV and MCHC (MCH and RDW were not reported). The decreased MCHC was also observed by Hu et al. (2008) at week 20 but not week 10 and attributed to RT-induced plasma osmolality changes and metabolic acidosis.

### Limitations

Firstly, blood samples were not obtained after the first and second bouts during weeks 1 and 12, and more frequently during the 12 weeks of RT (e.g., adding week 6). The consideration was to minimize blood sampling frequency while fulfilling the research aim of examining the acute and chronic responses to multiple bouts of RT in groups of differing recovery period without overcomplicating the analyses with too many time points. Secondly, like most training studies, we did not blind the investigators and were unable to control for diet and physical activity external to the sessions by providing standardized meals or having participants confined to the laboratory during the entire study. However, to reduce potential bias and interrater variability, the same team of research staff evaluated and trained both groups, and both groups of participants were encouraged to perform their best during training and evaluations. In addition, the monitoring and use of diet and physical activity as covariates strengthened the internal validity of the study. In fact, few studies utilized measures to such extent to control for confounding factors. Lastly, only males were recruited in the study as menstrual cycles could affect several outcome measures. However, there were no sex differences in strength and body composition adaptations to 7 weeks of 4 C or 3 NC days per week of RT (Hunter, 1985).

## Conclusion

The intent of this study is to provide impartial evidence on the effects of RT recovery period on various physiological variables and enable us to refine physical activity guidelines and exercise prescription. The results indicated similar improvements in strength for all five exercises and body composition, and changes in RBC parameters after 3 C or NC days of RT per week for 12 weeks. These findings refute current guidelines concerning the recovery period between RT sessions to optimize strength and muscle gains. C RT does not mean no rest as there is a 24 h recovery period between sessions and when performed 2–3 times a week, there is a rest of 4–5 days between weekly cycles. Moreover, most RBC parameters returned to baseline at 24 h post-3rd RT. Therefore, collectively, it is plausible that a recovery of 24–72 h between sessions did not matter as suggested by the results. There are no differential responses between C and NC RT to recommend one over the other for young, healthy individuals. For these individuals who perform 2–3 C days of RT per week, such as weekend warriors due to time constraints, they should not hold back for fear of inferior or detrimental adaptations if weekly volume and intensity are appropriate.

## Author Contributions

YY and JG designed the study. YY, PB, YW, JH, and HT contributed to data acquisition and analyses. YY, PB, and YW contributed to data interpretation and manuscript drafting. All authors contributed to the critical revision of the manuscript for important intellectual content and final approval of the version to be published.

## Conflict of Interest Statement

Theauthors declare that the research was conducted in the absence of any commercial or financial relationships that could be construed as a potential conflict of interest.

## Abbreviations

AT: aerobic training
BMC: bone mineral content
BMD: bone mineral density
C: consecutive
CV: coefficient of variance
DXA: dual-energy X-ray absorptiometry
Hb: hemoglobin
Hct: hematocrit
MCH: mean corpuscular hemoglobin
MCHC: mean corpuscular hemoglobin concentration
MCV: mean corpuscular volume
MVPA: moderate-vigorous physical activity
NC: non-consecutive
PV: plasma volume
RBC: red blood cell
RDW: red blood cell distribution width
RM: repetition maximum
RT: resistance training.
