# Supplementary material for: Effects of Consecutive Versus Non-consecutive Days of Resistance Training on Strength, Body Composition, and Red Blood Cells
Source: Front Physiol. 2018 Jun 18;9:725. doi: 10.3389/fphys.2018.00725 (PMC6015912; doi:10.3389/fphys.2018.00725)
Supplement: Supplementary file 1 [file Table_1.docx]

Supplementary Material

No Differential Responses between Consecutive and Nonconsecutive Days of Resistance Training on Strength, Body Composition and Red Blood Cells

**Yifan Yang^*^, Pang Boon Bay, Yongtai Raymond Wang, Junli Huang, Hilary Wei Jian Teo, Jorming Goh**

*** Correspondence:** Yifan Yang: yifan.yang@nie.edu.sg

**Supplementary Table S1**. Descriptive data of red blood cell parameters before resistance training (RT) at first session (Pre), and 0 and 24 h Post-3rd RT session in untrained (UT) and trained (T) states.

|  | **C (*n* = 15)** | | **NC (*n* = 15)** | | **Normal clinical** |
| --- | --- | --- | --- | --- | --- |
| **Variable** | **Mean (SD)** | **Range** | **Mean (SD)** | **Range** | **reference** |
| **RBC (x10^12^·L^-1^)** |  |  |  |  | 4.30-5.70 |
| UT Pre | 5.19 (0.28) | 4.68-5.60 | 5.33 (0.65) | 4.69-6.72 |  |
| UT 0 h Post-3rd | 5.20 (0.28) | 4.71-5.79 | 5.41 (0.65) | 4.53-6.54 |  |
| UT 24 h Post-3rd | 5.02 (0.24) | 4.68-5.52 | 5.22 (0.60) | 4.39-6.50 |  |
| T Pre | 5.17 (0.23) | 4.74-5.57 | 5.33 (0.60) | 4.66-6.61 |  |
| T 0 h Post-3rd | 5.23 (0.25) | 4.90-5.77 | 5.35 (0.58) | 4.68-6.60 |  |
| T 24 h Post-3rd | 5.11 (0.24) | 4.75-5.52 | 5.27 (0.67) | 4.50-6.74 |  |
| **RBC corr (x10^12^·L^-1^)** |  |  |  |  | 4.30-5.70 |
| UT Pre | 5.19 (0.28) | 4.68-5.60 | 5.33 (0.65) | 4.69-6.72 |  |
| UT 0 h Post-3rd | 5.11 (0.32) | 4.56-5.52 | 5.26 (0.75) | 4.51-7.10 |  |
| UT 24 h Post-3rd | 5.33 (0.41) | 4.57-6.09 | 5.43 (0.85) | 4.59-7.71 |  |
| T Pre | 5.17 (0.23) | 4.74-5.57 | 5.33 (0.60) | 4.66-6.61 |  |
| T 0 h Post-3rd | 5.12 (0.29) | 4.55-5.61 | 5.32 (0.65) | 4.46-6.66 |  |
| T 24 h Post-3rd | 5.20 (0.28) | 4.64-5.71 | 5.38 (0.61) | 4.57-6.54 |  |
| **Hb (g·dL^-1^)** |  |  |  |  | 12.9-17.0 |
| UT Pre | 15.2 (0.9) | 13.7-17.0 | 14.4 (1.2) | 12.1-16.3 |  |
| UT 0 h Post-3rd | 15.4 (0.8) | 14.4-16.7 | 14.7 (1.2) | 12.2-16.8 |  |
| UT 24 h Post-3rd | 14.8 (0.9) | 13.6-16.7 | 14.2 (1.1) | 12.4-15.9 |  |
| T Pre | 15.2 (0.8) | 14.0-16.9 | 14.4 (1.2) | 12.6-16.3 |  |
| T 0 h Post-3rd | 15.3 (0.8) | 14.0-16.7 | 14.4 (1.1) | 12.4-16.1 |  |
| T 24 h Post-3rd | 15.0 (0.8) | 13.6-16.3 | 14.3 (1.0) | 12.8-15.9 |  |
| **Hct (%)** |  |  |  |  | 37.5-49.3 |
| UT Pre | 43.2 (1.9) | 40.3-46.4 | 42.0 (2.9) | 36.5-46.6 |  |
| UT 0 h Post-3rd | 43.9 (1.6) | 41.2-46.6 | 43.0 (2.9) | 37.1-48.2 |  |
| UT 24 h Post-3rd | 41.9 (2.0) | 39.1-46.3 | 41.3 (2.3) | 37.5-45.0 |  |
| T Pre | 43.3 (1.7) | 40.6-46.6 | 42.3 (2.4) | 37.7-46.6 |  |
| T 0 h Post-3rd | 44.1 (1.4) | 41.7-46.8 | 42.8 (2.5) | 38.1-46.4 |  |
| T 24 h Post-3rd | 42.9 (1.7) | 39.7-45.9 | 41.8 (1.9) | 38.6-44.7 |  |
| **MCV (fL)** |  |  |  |  | 80.0-95.0 |
| UT Pre | 83.2 (2.6) | 78.5-87.4 | 80.0 (10.9) | 57.5-90.1 |  |
| UT 0 h Post-3rd | 84.4 (2.9) | 78.9-88.8 | 80.7 (11.2) | 58.0-92.0 |  |
| UT 24 h Post-3rd | 83.6 (2.7) | 78.4-88.6 | 80.1 (10.8) | 58.3-89.9 |  |
| T Pre | 83.8 (2.8) | 78.5-88.6 | 80.5 (10.8) | 57.0-89.2 |  |
| T 0 h Post-3rd | 84.4 (2.8) | 78.5-89.0 | 81.2 (11.1) | 57.7-90.2 |  |
| T 24 h Post-3rd | 84.0 (3.1) | 77.9-90.2 | 80.6 (10.8) | 57.3-89.9 |  |
| **MCH (pg)** |  |  |  |  | 27.0-33.0 |
| UT Pre | 29.4 (1.3) | 27.6-32.5 | 27.4 (4.2) | 19.1-31.5 |  |
| UT 0 h Post-3rd | 29.6 (1.3) | 27.8-32.7 | 27.5 (4.3) | 19.1-31.2 |  |
| UT 24 h Post-3rd | 29.5 (1.3) | 27.9-32.8 | 27.6 (4.3) | 19.2-31.4 |  |
| T Pre | 29.3 (1.2) | 27.5-32.3 | 27.5 (4.3) | 19.1-31.3 |  |
| T 0 h Post-3rd | 29.3 (1.5) | 27.2-32.6 | 27.4 (4.3) | 18.8-31.0 |  |
| T 24 h Post-3rd | 29.4 (1.3) | 27.2-32.3 | 27.6 (4.4) | 19.0-31.6 |  |
| **MCHC (g·dL^-1^)** |  |  |  |  | 32.0-36.0 |
| UT Pre | 35.3 (0.9) | 34.0-37.5 | 34.1 (0.9) | 32.1-35.6 |  |
| UT 0 h Post-3rd | 35.0 (1.0) | 33.6-36.9 | 34.1 (0.9) | 31.9-35.3 |  |
| UT 24 h Post-3rd | 35.3 (0.8) | 33.8-37.0 | 34.3 (1.0) | 32.2-35.7 |  |
| T Pre | 35.0 (1.0) | 33.6-37.0 | 34.0 (1.0) | 31.8-35.2 |  |
| T 0 h Post-3rd | 34.7 (1.0) | 33.5-37.0 | 33.7 (1.0) | 31.3-34.7 |  |
| T 24 h Post-3rd | 35.1 (0.9) | 34.0-36.6 | 34.1 (1.3) | 31.6-35.8 |  |
| **RDW (%)** |  |  |  |  | 11.4-14.8 |
| UT Pre | 12.5 (0.6) | 11.1-13.7 | 13.6 (1.4) | 12.1-16.0 |  |
| UT 0 h Post-3rd | 12.6 (0.7) | 11.0-14.1 | 13.7 (1.5) | 12.3-16.5 |  |
| UT 24 h Post-3rd | 12.6 (0.7) | 11.1-13.8 | 13.5 (1.3) | 12.2-15.7 |  |
| T Pre | 12.5 (0.7) | 11.3-13.9 | 13.5 (1.3) | 12.1-15.8 |  |
| T 0 h Post-3rd | 12.6 (0.7) | 11.4-13.9 | 13.5 (1.3) | 12.4-16.0 |  |
| T 24 h Post-3rd | 12.5 (0.7) | 11.3-13.8 | 13.4 (1.3) | 12.2-15.9 |  |
| *C, consecutive group; NC, nonconsecutive group; RBC, red blood cells; corr, correction for plasma volume change; Hb, hemoglobin; Hct, hematocrit; MCV, mean corpuscular volume; MCH, mean corpuscular hemoglobin; MCHC, mean corpuscular hemoglobin concentration; RDW, red blood cell distribution width.* | | | | | |
